# Long-term follow-up of coronary MR and CT angiography for prediction of cardiac events

**DOI:** 10.1186/1532-429X-16-S1-M2

**Published:** 2014-01-16

**Authors:** Adelina Doltra, Ashraf Hamdan, Alexander Huppertz, Eckart Fleck, Sebastian Kelle

**Affiliations:** 1 Internal Medicine/Cardiology, German Heart Institute Berlin, Berlin, Germany; 2 Cardiology, Heart Center, Chaim Sheba Medical Center, Tel Hashomer, Sackler Faculty of Medicine, Tel Aviv University, Tel Aviv, Israel; 3 Radiology, Imaging Science Institute Berlin, Charité Berlin, Berlin, Germany

## Background

Although some studies have shown the potential utility of coronary magnetic resonance angiography (CMRA) for the detection of coronary artery disease (CAD), almost no information exists regarding its prognostic value. Aim of our study was to compare the predictive value of CMRA and coronary multislice computed tomography angiography (CCTA) for prediction of cardiovascular events at long-term follow-up, with invasive coronary angiography (ICA) as the reference method.

## Methods

110 patients referred to MR for suspected or known CAD were prospectively included. A CMRA at 3T, a 64-slice CCTA and an ICA were performed in all patients. A luminal reduction ≥50% was defined as a significant stenosis. All patients were followed up for the occurrence of cardiovascular events, which comprised cardiovascular death, non-fatal myocardial infarction, necessity of revascularization and hospitalization due to cardiac events.

## Results

**Figure 1:**
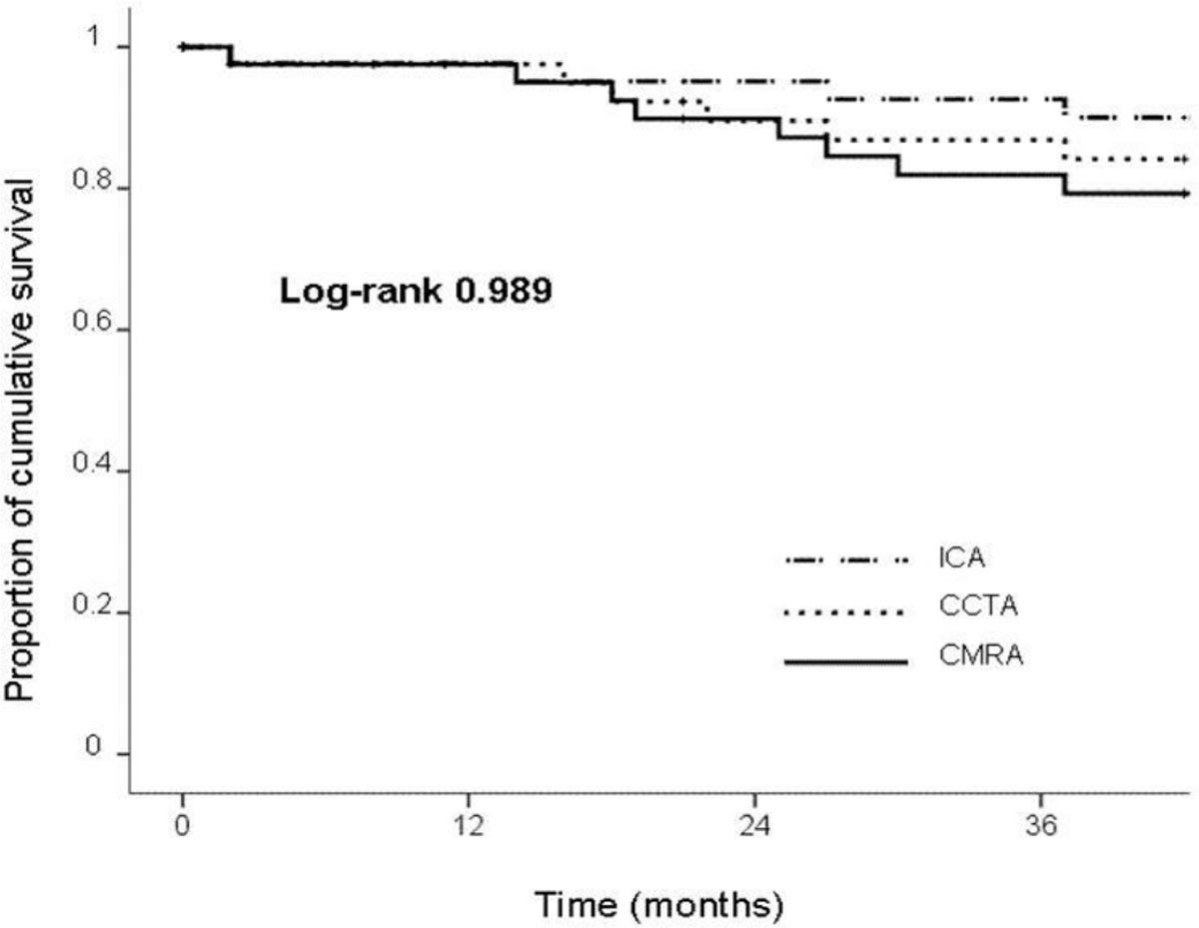
**Kaplan-Meier curve in patients without significant lesions in coronary magnetic resonance angiography (CMRA), coronary multislice computed tomography angiography (CCTA) and invasive coronary angiography (ICA)**. No significant differences in outcome are observed.

38 events were recorded during a mean follow-up of 40 ± 16 months. No significant differences in event-free survival were observed between the three techniques in the survival analysis (log-rank test p = 0.989) (Figure 1). The area under the receiver-operator curve was 65% (95% CI 55-76, p = 0.009) for CMRA, 70% (95% CI 60-80, p = 0.001) for CCTA and 75% (95% CI 66-85, p < 0.0001) for ICA.

## Conclusions

When assessing long-term prognosis, CMRA and CCTA demonstrated a similar prognostic value compared to invasive coronary angiography for prediction of cardiac events. CMRA has a non-inferior prognostic value compared to CCTA.

## Funding

None.

